# Shaping early childhood in the age of AI: holistic competencies, pedagogical practices, and ecological support

**DOI:** 10.3389/fpsyg.2026.1995020

**Published:** 2026-10-21

**Authors:** Li Pei

**Affiliations:** School of Education, Deakin University, Geelong, VIC, Australia

**Keywords:** AI, competencies, early childhood education, ecological systems, learning goals, teaching

## Abstract

The rapid development of artificial intelligence (AI) is reshaping the learning and developmental contexts in which young children grow, learn, and interact. Guided by an ecological perspective, this systematic conceptual mapping review examines 86 resources to explore what young children should learn and how they should be taught. Four themes were identified through thematic analysis: (1) holistic competencies for young children, encompassing foundational cognitive and social–emotional skills, physical and mental well-being, executive functions, learning dispositions, and AI literacy; (2) governance for AI integration in early childhood education; (3) professional development and systemic support for educators; and (4) play-based learning and human-centered approaches in AI-enhanced contexts. The findings suggest that thriving in the AI age requires moving beyond isolated digital skills toward holistic development, supported by a cohesive infrastructure that links classroom practice with structural governance. These findings highlight the necessity of cultivating holistic competencies beyond basic AI literacy in curriculum design, preparing children to navigate an ever-changing digital world. Furthermore, they underscore the imperative for robust institutional support, particularly through participatory research to support age-appropriate pedagogy and educators’ digital confidence, while highlighting the value of playful early learning. Concurrently, these insights establish a theoretical framework for future empirical research, offer actionable strategies for ethical child-centred AI implementation, and provide strategic directions for policy and practice to address the critical demand for transformative knowledge in early childhood education.

## Introduction

AI, resulting from rapidly developing technology, has brought both surprise and reflection regarding human intelligence (Yan et al., 2024; Lizarraga et al., 2025). Language models have evolved from early statistical methods to sophisticated neural networks: traditional AI relies on hand-crafted rules for specific tasks, whereas in the generative AI boom marked by ChatGPT’s release, models use attention mechanisms to learn contextual relationships from massive data. These models are still fundamentally statistical predictors, yet this mechanism enables emergent capabilities that resemble understanding (Bender et al., 2021; Lizarraga et al., 2025). It prompts us to reflect on which core competencies should be cultivated in the next generation to help them thrive in an uncertain world. Children are active but context-dependent learners, and early childhood experiences with AI-supported education may have long-term implications across the lifespan (Berk, 2018). AI cannot replace children’s construction of knowledge because learning must be based on active exploration and direct engagement with the environment (Piaget and Inhelder, 1969; Holmes et al., 2019). Children’s development is shaped through continuous interactions with their surrounding environments (Vygotsky, 1978; Holmes et al., 2019); therefore, understanding how education systems can more effectively support children’s development requires a holistic and systemic perspective. Greater attention is needed to understand how AI is influencing both teaching and learning in early childhood (0 to 8 years) (Miao and Holmes, 2023; Luo et al., 2024a).

One trending topic is about AI literacy. Huang et al. (2026, p.6) synthesised 102 systematic reviews about AI in education and pointed out AI literacy as “the ability to know and understand AI, use and apply AI, evaluate and create AI, and AI ethics” (Ng et al., 2021). Organizations such as UNESCO, OECD, and Digital Promise are developing frameworks that emphasize the knowledge, skills, attitudes, ethical understanding, and responsible use of AI technologies to support educational transformation (Digital Promise, 2024; OECD and European Commission, 2026; UNESCO, 2024). While research and policies have increasingly mapped AI literacy in primary and secondary education, its conceptualization and implementation in early childhood education require more attention (Luo et al., 2024a; Su and Zhong, 2022; Su et al., 2023). Beyond cognitive competencies, early childhood education also plays a critical role in shaping children’s psychosocial development, which serves as the foundation for lifelong development, including autonomy, competence, and confidence (Erikson, 1950; Berk, 2018).

However, AI literacy itself is not sufficient (Atias and Mawasi, 2025) to face the challenge brought by rapidly technology on education. A comprehensive redesign of what to learn and how to teach to meet the demands of the AI era is significant (Holmes et al., 2019; Pei, 2025a). More importantly, education should not simply adapt to the challenges posed by technological advancements. Rather, it should take a proactive role in shaping the future, guiding technological development, and equipping the next generation with the competencies needed to navigate an increasingly uncertain world. This perspective aligns with Luckin et al. (2016), who metaphorically described the evolving relationship between education and technology as a continuing race, emphasizing the persistent tension and accelerating pace of innovation between the two domains. AI literacy may help children understand how to use AI, but it does not fully address how children can live, learn, and thrive in an uncertain world shaped by AI and rapid technological change.

In consideration of what competencies our society really needs, the World Economic Forum (2025) emphasizes the importance of nurturing both technological awareness and human-centered qualities, and highlights a set of core competencies increasingly in demand across industries. Meanwhile, growing scholarly attention has been given to ensuring that AI serves as humanity’s ally rather than undermining human intelligence (Yang et al., 2024; Chacon and Langer, 2026), which suggests that competencies beyond AI literacy warrant greater attention in preparing young children for an AI-driven future (Pei, 2025a).

For example, Theory of Mind is a core component of human intelligence that enables individuals to attribute mental states such as beliefs, desires, intentions, and knowledge to themselves and others (Premack and Woodruff, 1978) and differs from large language model–based AI systems, which are often argued to lack grounded understanding and instead rely on pattern-based language learning rather than genuine comprehension (Bender et al., 2021; Lizarraga et al., 2025). In relation, executive functions, including working memory (the ability to hold and manipulate information over short periods), inhibitory control (the capacity to suppress impulsive or inappropriate responses), and cognitive flexibility (the ability to shift attention and adapt to changing demands), help young children regulate their thoughts, emotions, and behaviors (Diamond, 2013, 2016; Ibbotson, 2023) when they face uncertainties and thus benefit cognitive and social–emotional competencies. On top of this, learning dispositions, which are a set of enduring tendencies and attitudes that shape how young children approach learning, provide young children with curiosity, persistence, and creativity (Carr and Claxton, 2002; Pei, 2025c). United Nations Children’s Fund (UNICEF) (2020) draft guidance emphasizes that AI for children must be designed and implemented with their rights, safety, and well-being as a priority, highlighting the need for ethical, inclusive, and developmentally appropriate approaches in policy and practice. These developments present pedagogical challenges as well, particularly for female educators, who comprise the majority of the early childhood workforce (UNESCO, 2023).

Accordingly, this study fills this gap through the lens of ecological systems theory (Bronfenbrenner, 1979, 2005). It systematically examines existing literature on the competencies children need in the context of AI-driven transformation, the challenges teachers face in fostering these competencies, and the forms of support required. In doing so, it aims to generate timely insights to inform innovation and quality improvement in early childhood education.

The research questions (RQs) are as follows:

*RQ1:* What core competencies are essential for young children to thrive in an AI-influenced world?

*RQ2:* How is AI influencing early childhood educators’ practice, and what supports are needed?

## Theoretical framework

The research questions in this study address the individual, pedagogical, and systemic dimensions of AI integration, aligning with Bronfenbrenner’s ecological systems theory (Bronfenbrenner, 1979, 2005). It emphasizes how children’s learning and development are shaped by multiple, interconnected environmental systems, from direct interactions with educators and peers to broader institutional and policy contexts, offering a holistic perspective for understanding AI’s impact on early education.

As shown in Figure 1, it allows the study to move beyond a narrow focus on technology or skills to examine how AI-related learning opportunities and challenges are distributed across the microsystem of direct child experiences, the mesosystem of family, school, and peer connections, the exosystem of institutional and community influences, the macrosystem of societal, cultural and policy contexts, and the chronosystem of temporal and developmental dynamics over time (Bronfenbrenner, 1979, 2005). Specifically, Children’s experiences are directly influenced by microsystem factors such as pedagogy (Bronfenbrenner, 1979; Piaget and Inhelder, 1969; Vygotsky, 1978). They learn from daily interaction with teachers, other individuals, and surrounding environments (Berk, 2018; Bronfenbrenner, 1979). Moreover, the interconnections between family, school, and peers at the mesosystem make an impact on how children directly experience the world (Berk, 2018; Bronfenbrenner, 1979, 2005). As such, indirect institutional and community contexts shape how teachers, schools, families, and peers interact with children at the microsystem level, as well as how family and school connect at the mesosystem level (Bronfenbrenner, 1979). On top of this, the societal, cultural, and policy contexts at the macrosystem level shape how individuals behave in the exosystem, mesosystem, and microsystem (Bronfenbrenner, 1979, 2005; Holmes et al., 2019; UNESCO, 2024). Finally, the chronosystem provides changes to all other system levels as time changes (Berk, 2018; Bronfenbrenner, 2005). This ecological perspective provides a holistic understanding of the conditions necessary for young children’s thriving, the evolving roles of educators, and the systemic supports required for developmentally appropriate AI integration.

**Figure 1:**
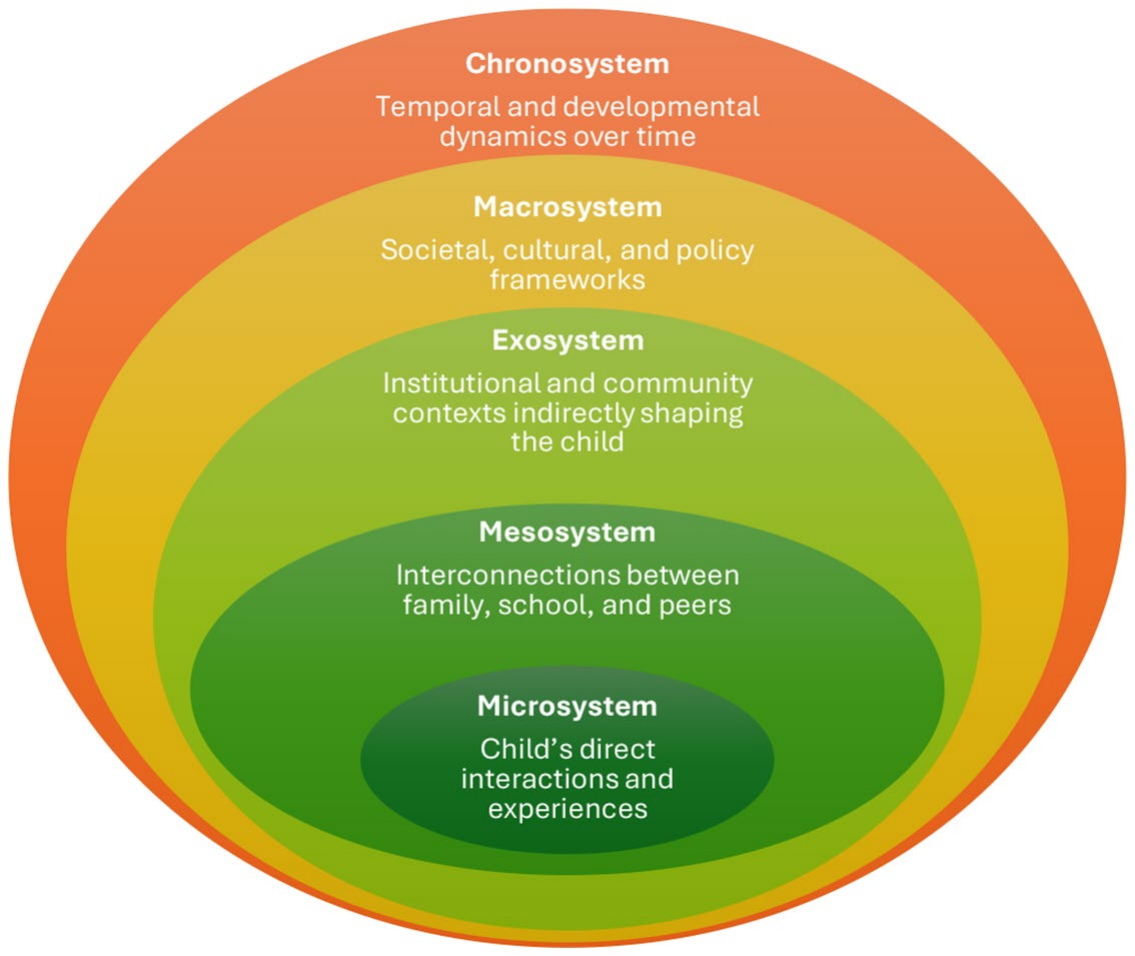
Conceptual framework: applying Bronfenbrenner (1979, 2005)’s ecological systems theory.

## Methods

### Data collection

From 27 May 2024 to 20 June 2026, a systematic literature search was conducted across academic databases, organizational repositories, and supplementary snowballing searches using search engines to ensure comprehensive coverage. Primary databases included APA PsycNet, ERIC, EBSCOhost, and Web of Science, while Google Scholar captured additional studies. Search terms addressed the intersection of technology, pedagogy, and educator demographics, with Boolean operators used to combine the following search terms: “AI” AND “education”; “AI” AND “early childhood”; “AI” AND “preschools”; “AI” AND “early childhood educators”; “AI” AND “competencies”; “competencies” AND “young children”; and “core skills of young children”.

To ensure a holistic understanding of the evolving AI landscape, this study also includes relevant key reports and policy documents from organizational sources (OECD iLibrary, UNESCO Digital Library, national education department websites, and Harvard reports), which are consistent with the methodological approach of ecological systems theory (Bronfenbrenner, 1979, 2005). Child development is understood as the result of dynamic interactions between the individual and multiple layers of environmental systems. It is shaped not only by learning and interactive contexts at the micro and meso levels, but also by broader meso- and macro-level factors such as institutional structures, policy frameworks, and sociocultural conditions, and is influenced by processes at the chronosystem.

Figure 2 presents the PRISMA-guided resource selection process (Page et al., 2021) for this systematic review. A total of 1,195 records were initially identified across all sources, including 1,006 from academic databases and 189 from organizational sources. After title and abstract screening, 118 resources were assessed for eligibility and initially categorised as: journal papers (*n* = 63, 1.1 to 1.63), books (*n* = 15, 2.1 to 2.15), and others (*n* = 40, 3.1 to 3.40). During the final screening, publications were excluded if they were not closely related to core competencies for young children (0 to 8 years old) in the AI age, AI-driven pedagogical changes, or support mechanisms for effective early childhood education (*n* = 29). Publications were also excluded due to article quality issues, such as lacking clear research methods or evidence supporting the findings (*n* = 2), and duplication (*n* = 1). Finally, this study included 86 resources in the thematic synthesis as listed in Table 1. Each has a unique reference number for mapping and analysis. These resources consist of (A) 26 empirical studies, (B) 28 conceptual and review papers, (C) 14 scholarly books, and (D) 18 reports and policy documents as presented in Table 2.

**Figure 2:**
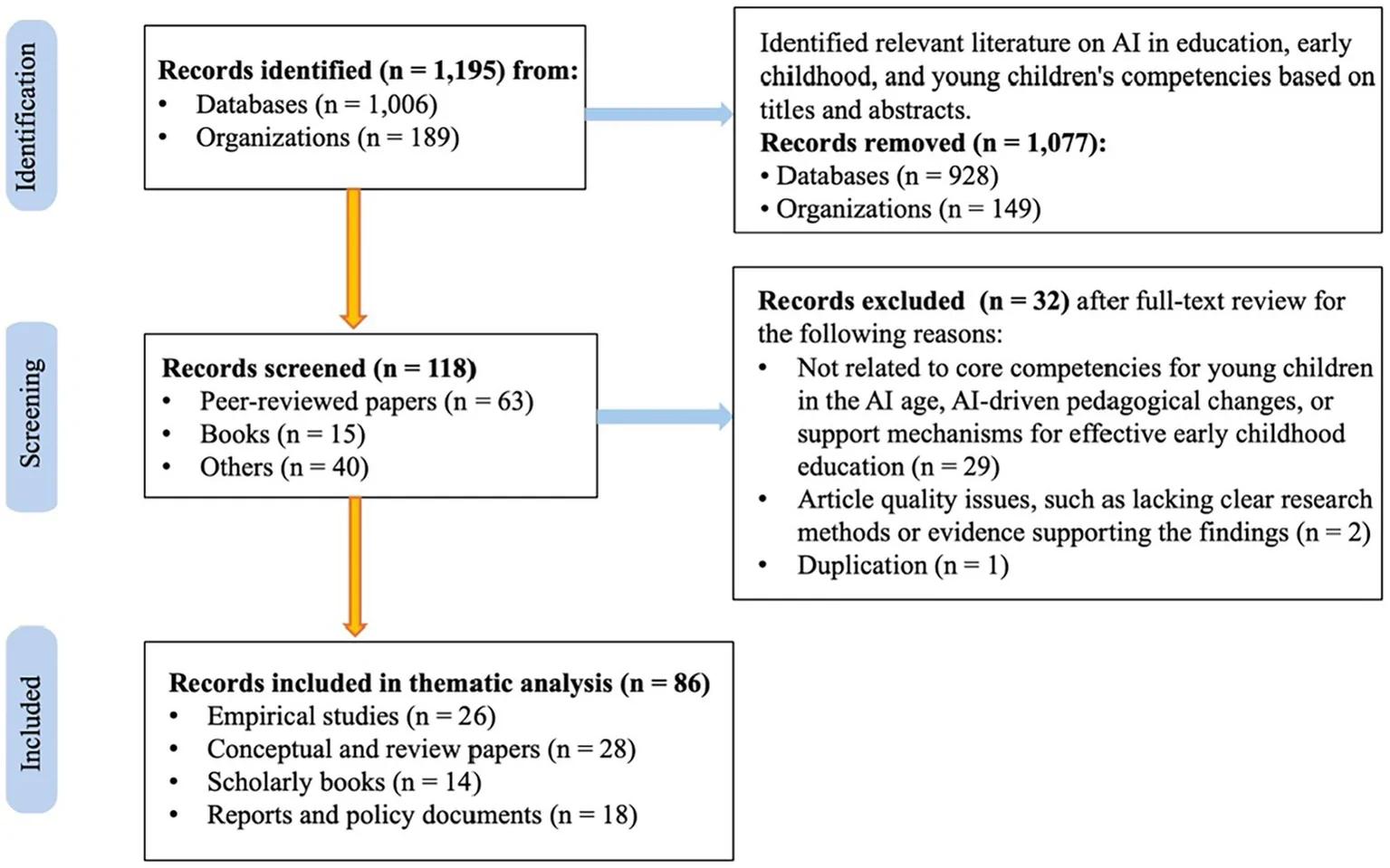
PRISMA flow [following Page et al. (2021)].

**Table 1:** Indexed reference number of the included 86 resources.

No.	Author/year	No.	Author/year	No.	Author/Year	No.	Author/Year
1	Huang et al. (2026)	23	Jiang et al. (2025)	45	Lizarraga et al. (2025)	67	Holmes et al. (2019)
2	Su et al. (2023)	24	Lim et al. (2023)	46	Bender et al. (2021)	68	Prentzas (2013)
3	Su and Zhong (2022)	25	Lim et al. (2023)	47	Singer and Lamm (2009)	69	Department of Education (2023)
4	Su and Yang (2022)	26	Lo (2023)	48	Moffitt et al. (2010)	70	Harvard University (2011)
5	Li et al. (2026)	27	Luo et al. (2024a)	49	Pei (2026)	71	Harvard University (2012)
6	Ng et al. (2023)	28	Luo et al. (2024b)	50	Ibbotson (2023)	72	Collett et al. (2022)
*7*	Gribiea (2026)	29	Pei (2025c)	51	Kölemen and Yıldırım (2025)	73	Department of Health and Human Services (2003)
8	Atias and Mawasi (2025)	30	Matthews et al. (2025)	52	Williams et al. (2019)	74	Department of Education, Employment and Workplace Relations (2009)
9	Ng et al. (2021)	31	Nguyen et al. (2023)	53	Kewalramani et al. (2021)	75	Digital Promise (2024, June 18)
10	Almatrafi et al. (2024)	32	Okonkwo and Ade-Ibijola (2021)	54	Lee (2026)	76	Luckin et al. (2016)
11	Adiguzel et al. (2023)	33	Pangrazio and Sefton-Green (2021)	55	Bendini and Devercelli (2022)	77	Ministry of Education (MOE) (2025, April 3)
12	Berson et al. (2025)	34	Roll and Wylie (2016)	56	Ben Mardell et al. (2023)	78	OECD (2023)
13	Carr and Claxton (2002)	35	Srinivasan (2022)	57	Diamond (2016)	79	OECD and European Commission. (2026)
14	Chassignol et al. (2018)	36	Su and Yang (2024)	58	Fleer (2021)	80	OECD (2025)
15	Chen et al. (2020)	37	Su and Yang (2023)	59	Gao and Huo (2017)	81	Office of Educational Technology (2023)
16	Chiu et al. (2023)	38	Su et al. (2024)	60	Lee (2025)	82	Stanford University (2024)
17	Collie and Martin (2025)	39	Willoughby et al. (2019)	61	Mhlanga (2023)	83	UNESCO (2023, November 8)
18	Diamond (2013)	40	Xu et al. (2024)	62	NAEYC (2022)	84	Miao and Holmes (2023)
19	Dresp-Langley (2020)	41	Yang (2022)	63	Selwyn (2019)	85	AI4K12 Initiative (2024)
20	Ghamrawi et al. (2024)	42	Yang et al. (2024)	64	Trilling and Fadel (2009)	86	Miao and Cukurova (2024)
21	Howard et al. (2021)	43	Yu et al. (2025)	65	Wellman (2014)		
22	Hwang et al. (2020)	44	Chacon and Langer (2026)	66	Griffin et al. (2016)		

**Table 2:** Distribution of included literature by methodological category.

Category	Reference numbers of included sources	Total number	Percentage (%)
Empirical papers (A)	3 (Su and Zhong, 2022), 5 (Li et al., 2026), 7 (Gribiea, 2026), 17 (Collie and Martin, 2025), 20 (Ghamrawi et al., 2024), 21 (Howard et al., 2021), 23 (Jiang et al., 2025), 24 (Lim et al., 2023), 27 (Luo et al., 2024a), 28 (Luo et al., 2024b), 29 (Pei, 2025c), 30 (Matthews et al., 2025), 36 (Su and Yang, 2024), 37 (Su and Yang, 2023), 38 (Su et al., 2024), 39 (Willoughby et al., 2019), 40 (Xu et al., 2024), 42 (Yang et al., 2024), 43 (Yu et al., 2025), 44 (Chacon and Langer, 2026), 48 (Moffitt et al., 2010), 49, Pei (2026), 51 (Kölemen and Yıldırım, 2025), 52 (Williams et al., 2019), 53 (Kewalramani et al., 2021), and 54 (Lee, 2026)	26	30.2
Conceptual and review papers (B)	1 (Huang et al., 2026), 2 (Su et al., 2023), 4 (Su and Yang, 2022), 6 (Ng et al., 2023), 8 (Atias and Mawasi, 2025), 9 (Ng et al., 2021), 10 (Almatrafi et al., 2024), 11 (Adiguzel et al., 2023), 12 (Berson et al., 2025), 13 (Carr and Claxton, 2002), 14 (Chassignol et al., 2018), 15 (Chen et al., 2020), 16 (Chiu et al., 2023), 18 (Diamond, 2013), 19 (Dresp-Langley, 2020), 22 (Hwang et al., 2020), 25 (Lim et al., 2023), 26 (Lo, 2023), 31 (Nguyen et al., 2023), 32 (Okonkwo and Ade-Ibijola, 2021), 33 (Pangrazio and Sefton-Green, 2021), 34 (Roll and Wylie, 2016), 35 (Srinivasan, 2022), 41 (Yang, 2022), 45 (Lizarraga et al., 2025), 46 (Bender et al., 2021), 47 (Singer and Lamm, 2009), and 50 (Ibbotson, 2023)	28	32.6
Scholarly books (C)	55 (Bendini and Devercelli, 2022), 56 (Ben Mardell et al., 2023), 57 (Diamond, 2016), 58 (Fleer, 2021), 59 (Gao and Huo, 2017), 60 (Lee, 2025), 61 (Mhlanga, 2023), 62 (NAEYC, 2022), 63 (Selwyn, 2019), 64 (Trilling and Fadel, 2009), 65 (Wellman, 2014), 66 (Griffin et al., 2016), 67 (Holmes et al., 2019), and 68 (Prentzas, 2013)	14	16.3
Reports and policy documents (D)	69 (Department of Education, 2023), 70 (Harvard University, 2011), 71 (Harvard University, 2012), 72 (Collett et al., 2022), 73 (Department of Health and Human Services, 2003), 74 (Department of Education, Employment and Workplace Relations, 2009), 75 (Digital Promise, 2024, June 18), 76 (Luckin et al., 2016), 77 (Ministry of Education MOE, 2025, April 3), 78 (OECD, 2023), 79 (OECD and European Commission., 2026), 80 (OECD, 2025), 81 (Office of Educational Technology, 2023), 82 (Stanford University, 2024), 83 (UNESCO, 2023, November 8), 84 (Miao and Holmes, 2023), 85 (AI4K12 Initiative, 2024), and 86 (Miao and Cukurova, 2024)	18	20.9
Total	N/A	86	100.0

The final selected literature evidence with reference numbers was systematically documented to ensure transparency and traceability, along with the initial classification process, the details of this process are provided in Appendix 1. The dataset spans international contexts, predominantly from the Asia-Pacific region, North America and Europe (including the United Kingdom), with additional representation from the Middle East, Africa, and South America.

### Data analysis

Data were analyzed using a combined deductive and inductive approach (Theophilus, 2018; Pei, 2025b), as shown in Figure 3. Step 1 involved literature familiarization, confirming included studies, and noting a general impression of each piece. Step 2 consisted of categorizing literature deductively using Ecological Systems Theory, allowing individual pieces to appear across multiple system levels, as illustrated in Figure 1. Step 3 focused on initial theme coding to find potential patterns in an inductive way, following Braun and Clarke (2006), emphasizing the generation of themes relevant to the research questions. Step 4 involved reviewing and validating themes, critically refining them, and generating additional themes as needed to ensure accuracy and relevance. Step 5 addressed theme integration, removing unnecessary literature, adding updated sources if required, and organizing final themes and subthemes under system levels. Step 6 comprised writing up the findings and synthesizing results into a coherent narrative. Together, these steps produced a comprehensive conceptual framework that maps literature evidence to support children’s development in the AI age within the ecological system landscape (Bronfenbrenner, 1979, 2005).

**Figure 3:**
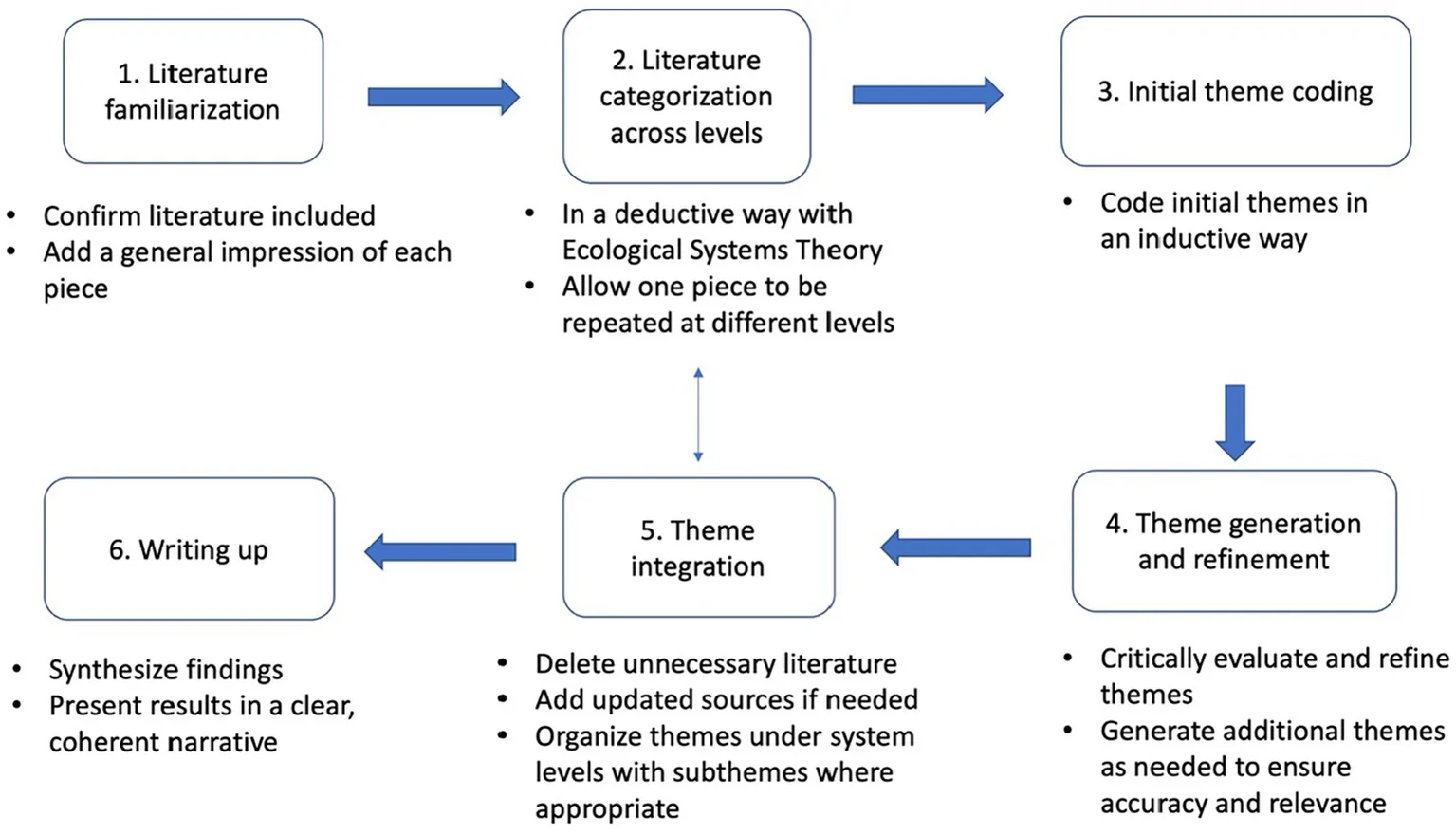
Thematic coding.

## Findings

The findings are organised to address two RQs and are interpreted through an ecological systems lens, spanning the chronosystem (temporal and developmental dynamics), macrosystem (societal, cultural, and policy contexts), exosystem (institutional and community influences), mesosystem (connections among family, school, and peers), and microsystem (children’s direct experiences). Four themes and their associated subthemes are presented in Table 3, supported by evidence from the reviewed literature.

**Table 3:** Mapping themes and subthemes identified through thematic analysis.

Theme	Subtheme	Coding number
Theme 1: Holistic competencies for young children (chronosystem & macrosystem)	Subtheme 1.1: Foundational Cognitive and Social–Emotional Skills	A 42 B 45, 46, 47 C 55, 62, 64, 65, 67 D 78, 79
	Subtheme 1.2: Physical and Mental Well-being in the Digital Age	A 30 B 19 D 80
	Subtheme 1.3: Executive Functions as a Foundation for Cognitive and Social–Emotional Competencies	A 21, 39, 44, 48 B 18, 45, 46, 50 C 57, 66 D 70, 71
	Subtheme 1.4: Learning Dispositions and Approaches to Learning	A 29 B 13 C 59, 66, 67 D 73, 74, 79, 80
	Subtheme 1.5: AI Literacy	A 3, 5, 28, 38 B 1, 2, 4, 5, 9, 10 D 79
Theme 2: Governance for AI integration in early childhood education (macrosystem)		A 40, 43 B 9, 25, 31, 32 C 61, 67 D 69, 76, 77, 79, 80, 81
Theme 3: Professional development and systemic support for educators (exosystem)	Subtheme 3.1: Shifting Professional Roles and Digital Confidence	A 7, 17, 20, 23, 24, 27, 51, 54 B 1, 6, 11, 25, 33 D 72, 75, 76, 83, 86
	Subtheme 3.2: Participatory Design for Professional Development	A 49, 51, 54 B 12 D 84
Theme 4: Play-based learning and human-centered in ai-enhanced contexts (mesosystem & microsystem)	Subtheme 4.1: Reimagining Play-Based Pedagogy in the AI Age	A 30, 37, 38, 49, 52, 53, 54 B 4, 8 C 55, 56, 58, 62, 68 D 82, 85
	Subtheme 4.2: AI as a Support for Teachers	A 3, 27, 36, 38, 42 B 14, 15, 16, 22, 26, 34, 35, 41 C 60, 62, 63

Empirical papers (A), conceptual and review papers (B), scholarly books (C), and reports and policy documents (D).

### Themes 1: Holistic competencies for young children (chronosystem and macrosystem)

#### Subtheme 1.1: Foundational cognitive and social–emotional skills

Foundational cognitive skills such as literacy and numeracy remain core areas of early years education quality (Bendini and Devercelli, 2022; NAEYC, 2022), providing a basis for understanding society amid rapid technological change. Social–emotional skills such as resilience, adaptability, motivation, and self-awareness are increasingly intertwined with cognitive development (Trilling and Fadel, 2009; OECD, 2023). Critical thinking and creativity are essential for identifying bias in AI-generated content and rethinking solutions to open-ended problems (OECD and European Commission, 2026), grounded in cognitive knowledge (Yang et al., 2024) and higher-order thinking (Holmes et al., 2019).

Early childhood is also a critical period for developing Theory of Mind, a social-cognitive competency that bridges cognitive and emotional understanding and supports empathy, perspective-taking, and related skills (Wellman, 2014; Singer and Lamm, 2009). Human understanding is deeply rooted in emotion and lived social experience, whereas AI systems rely on probabilistic pattern matching without grounded understanding or lived experience (Bender et al., 2021; Lizarraga et al., 2025). Theory of Mind thus highlights a key distinction between human intelligence and large language model–based AI systems.

#### Subtheme 1.2: Physical and mental well-being in the digital age

Children’s physical and mental well-being faces emerging challenges in the digital age, particularly in relation to visual health. Excessive screen time has been associated with increased risk of myopia, largely due to reduced exposure to outdoor light (Dresp-Langley, 2020). OECD (2025) further highlights that prolonged screen exposure is linked not only to eye strain and myopia but also to broader impacts on sleep quality, physical activity, and mental health, including exposure to inappropriate content and online safety risks.

Collectively, the literature suggests that digital technologies introduce a multi-dimensional risk profile for children’s well-being that extends beyond physical health to psychological and behavioural domains. Consequently, safeguarding children’s well-being requires a multi-sectoral response involving digital service providers, health professionals, educators, parents, and children to construct a safer digital ecology. In response, Matthews et al. (2025) propose an embeddable approach that reduces screen exposure through physical tools with digital functions, offering a potential strategy for supporting healthier developmental conditions in the AI era.

#### Subtheme 1.3: Executive functions as a foundation for cognitive and social–emotional competencies

Executive functions (EFs) are increasingly critical in the context of AI systems that generate fluent and persuasive yet potentially biased information embedded in training data (Bender et al., 2021; Chacon and Langer, 2026; Lizarraga et al., 2025). In such environments, EFs underpin critical thinking, creativity, collaboration, and ethical reasoning by enabling children to evaluate complex information, generate novel ideas, and engage in reflective decision-making (Diamond, 2013; Ibbotson, 2023).

EFs comprise three core components: working memory, inhibitory control, and cognitive flexibility, with cognitive flexibility emerging from the coordinated interaction of the former two. Together, these functions support higher-order processes such as reasoning, problem solving, and planning (Diamond, 2013, 2016). Conceptualised as an “air traffic control system” that coordinates attention and behaviour, EFs are biologically grounded yet environmentally shaped, developing through interaction, play, and contextual learning (Harvard University, 2011, 2012; Griffin et al., 2016).

Evidence consistently shows that EFs are strongly associated with academic achievement, self-regulation, social functioning, mental health, and long-term life outcomes beyond early intelligence and performance measures (Diamond, 2016; Willoughby et al., 2019; Howard et al., 2021; Ibbotson, 2023). For example, early inhibitory control has been linked to adult health, income, criminal behaviour, and subjective well-being in a 32-year longitudinal study of over 1,000 children (Moffitt et al., 2010; Diamond, 2016). Early childhood, particularly between ages 3 and 5, represents a sensitive period of rapid EF development, making early cultivation especially significant given its long-term implications for cognitive and socio-emotional outcomes (Harvard University, 2012; Diamond, 2016).

#### Subtheme 1.4: Learning dispositions and approaches to learning

Learning dispositions and approaches to learning are interchangeable terms describing young children’s characteristic engagement with learning(Pei, 2025c; Carr and Claxton, 2002; Gao and Huo, 2017). In the AI age, they are essential for adaptive engagement with technology, flexibility, and collaboration in complex digital environments.

Defined as “enduring habits of mind and actions, and tendencies to respond in characteristic ways to situations, for example, maintaining an optimistic outlook, being willing to persevere, approaching new experiences with confidence” (Department of Education, Employment and Workplace Relations, 2009, p. 10), learning dispositions are emphasised in early childhood education, particularly in Australia and New Zealand. In other contexts such as the U.S. and China, they are termed “approaches to learning,” including curiosity, persistence, and creativity (Pei, 2025c; Department of Health and Human Services, 2003; Gao and Huo, 2017).

These dispositions shape children’s engagement with uncertain AI-related learning conditions. They are underpinned by physical and mental well-being (OECD, 2025; OECD and European Commission, 2026) and executive functions (Griffin et al., 2016), while also being shaped by sociocultural contexts and pedagogical practices (Holmes et al., 2019). Thus, learning dispositions operate as a mediating layer between internal capacities and external environments, making how children learn increasingly central in the AI age.

#### Subtheme 1.5: AI literacy

Emerging scholarship highlights the importance of embedding foundational AI literacy (Almatrafi et al., 2024; Huang et al., 2026) in early childhood curricula, enabling children to critically evaluate AI outputs and understand basic concepts such as machine learning (Ng et al., 2021). Early AI education has been shown to support children’s computational thinking, sequencing, self-regulation, and theory of mind (Su et al., 2024).

AI literacy has been conceptualised as comprising three core competencies: AI knowledge, skills, and attitudes (Su and Zhong, 2022), aligning with OECD and European Commission (2026), while Luo et al. (2024b) extend this to five dimensions: Safety, Identity, Attitude, Cognition, and Capability (SIACC). Both frameworks reflect the broadened definition of AI literacy in education proposed by Ng et al. (2021) and Huang et al. (2026, p.6). However, these models are underpinned by broader developmental competencies discussed in previous subthemes. AI knowledge draws on foundational cognitive abilities, while attitudes and capabilities such as creativity and critical thinking are closely linked to executive functions and learning dispositions, including working memory, inhibitory control, cognitive flexibility, curiosity, and persistence.

In practice, even children as young as four can grasp basic AI concepts and apply them creatively, with older preschoolers demonstrating more complex and interactive forms of AI-related creativity (Su and Yang, 2024). While earlier work focused on defining AI literacy frameworks and identifying implementation challenges in early childhood education (Su et al., 2023; Su and Zhong, 2022), recent developments have shifted toward integrating large language models as interactive assessment partners to enhance systemic educational quality (Li et al., 2026).

### Theme 2: Governance for AI integration in early childhood education (macrosystem)

In response to the growing use of AI in education, national and international policy frameworks have emerged to guide governance for AI integration in early childhood education. For example, Australia’s Department of Education (2023) released the “Australian Framework for Generative Artificial Intelligence in Schools”, outlining principles of pedagogy, wellbeing and ethical use, transparency, equity and inclusion, and collaboration. Similarly, China’s Ministry of Education (MOE) (2025) set priorities for the 2025 Digital Education Strategic Action Plan, emphasizing AI literacy development for educational administrators and school leaders. The U.S. Office of Educational Technology (2023) stresses a people-centred approach involving students, educators, and families, while OECD (2025) highlights the need for child-centred governance addressing privacy, bias, and equity.

Across these frameworks, governance is increasingly oriented toward balancing innovation with ethical safeguards. Robust data privacy protection is identified as essential for building trust in AI systems (Nguyen et al., 2023), while transparent communication of AI capabilities supports responsible use and lifelong learning (Mhlanga, 2023). Although AI can enhance personalization, teaching quality, and equity (Luckin et al., 2016), restrictive approaches such as banning generative AI may be counterproductive, potentially increasing unregulated use (Lim et al., 2023). This has led to a growing emphasis on governance models that integrate innovation with regulation, ensuring equitable access, accountability, and basic AI understanding (Ng et al., 2021; Nguyen et al., 2023; Okonkwo and Ade-Ibijola, 2021).

In early childhood contexts, governance challenges are further intensified by children’s developmental characteristics. Young children often perceive robots and AI characters as social agents (Prentzas, 2013; Xu et al., 2024), raising concerns about over-trust and blurred human–machine boundaries. This highlights the need for developmentally appropriate design that supports healthy social development and accurate understanding of technology (OECD and European Commission, 2026), particularly given concerns about age bias and the underrepresentation of young children’s voices in AI systems (Digital Promise, 2024). Accordingly, emerging scholarship emphasizes the importance of child-specific datasets, algorithmic transparency, and strong ethical governance to safeguard young learners (Yu et al., 2025).

### Theme 3: Professional development and systemic support for educators (exosystem)

#### Subthemes 3.1: Shifting professional roles and digital confidence

The evolving educational landscape is reshaping the roles of early childhood educators. With female educators comprising 94% of pre-primary teachers worldwide (UNESCO, 2023), they face the dual challenge of integrating AI into practice while addressing digital literacy gaps (Ng et al., 2023) and workplace stress (Collett et al., 2022; Digital Promise, 2024). Although AI may reduce workload in curriculum design and material preparation (Collie and Martin, 2025), strengthening educators’ digital confidence (Pangrazio and Sefton-Green, 2021) and critical engagement is more important than superficial technical training (Lee, 2025). However, research on teachers’ AI literacy remains limited (Huang et al., 2026), despite growing policy attention to AI competencies for teachers (Miao and Cukurova, 2024).

AI-enabled adaptive systems can personalise learning and improve teaching processes (Adiguzel et al., 2023; Lim et al., 2023; Luckin et al., 2016), but require teacher oversight to contextualise outputs and address socioemotional needs. The literature consistently emphasises human–AI collaboration (Jiang et al., 2025), while over-automation risks narrowing educators’ professional roles (Ghamrawi et al., 2024; Selwyn, 2019; Luo et al., 2024a). Thus, educators are expected to critically evaluate AI-generated resources and adapt them to pedagogical contexts (Miao and Cukurova, 2024).

Some educators express concerns that AI use in early childhood may be inappropriate due to privacy and ethical risks, highlighting the need to strengthen technological self-efficacy and well-being (Kölemen and Yıldırım, 2025). Accordingly, professional development initiatives and pre-service training focused on digital literacy and self-efficacyare critical for supporting AI integration and enhancing educators’ digital confidence (Gribiea, 2026; Lim, 2023; Ng et al., 2023; Pangrazio and Sefton-Green, 2021).

#### Subthemes 3.2: Participatory design for professional development

Effective AI integration in early childhood education requires evidence-based professional development for teachers (Miao and Holmes, 2023). Early childhood educators are eager for training that integrates appropriate pedagogy with AI applications (Kölemen and Yıldırım, 2025), while early childhood education remains strongly context-based, with young children learning through play and lived experiences (Pei, 2026).

Beyond online training, involving educators as co-designers of AI integration can strengthen their AI literacy and digital confidence, while ensuring developmental appropriateness and local values are embedded in practice and informing policy development (Berson et al., 2025; NAEYC, 2022). Design-based research (DBR) further demonstrates how co-design enhances teacher agency, enabling educators to shape both conceptual and practical dimensions of ethical and developmentally appropriate AI interventions (Lee, 2026).

### Theme 4: Play-based learning and human-centered in AI-enhanced contexts (mesosystem and microsystem)

#### Subtheme 4.1: Reimagining play-based pedagogy in the AI age

Appropriate technology adoption may enhance early learning when it complements hands-on, socially interactive experiences and preserves children’s autonomy (Bendini and Devercelli, 2022; Stanford University, 2024; Atias and Mawasi, 2025). High-quality early childhood education fosters development through open-ended play, storytelling, collaboration, and exploration (Fleer, 2021). Thematic mapping further supports teachers in designing culturally responsive learning aligned with children’s interests through local cultural integration (Pei, 2026). Accordingly, AI tools should support rather than replace human learning processes, raising not only what AI can do, but what it should do in early learning.

Early childhood learning is grounded in relationships, play, and care (NAEYC, 2022). Play-based approaches, such as block-building apps for sustainable city design, connect STEM learning with creative problem-solving (Ben Mardell et al., 2023). Computational thinking can be developed through embodied, tangible, and unplugged activities, highlighting the importance of appropriate digital tool design (Matthews et al., 2025). This pedagogical foundation also supports early AI concept learning (Lee, 2026), with embodied play and guided dialogue aligned with AI4K12 “Big Ideas” and developmentally appropriate practice (AI4K12 Initiative, 2024; NAEYC, 2022).

AI-enabled role-play and robotic systems have been shown to support children’s learning (Su and Yang, 2022; Kewalramani et al., 2021), including inquiry literacy through interactive play. Prior work has identified diverse educational robots, including humanoid, social, and mobile systems (Prentzas, 2013). The PopBots curriculum demonstrated that young children can develop AI understanding through interaction and programming, though with varying conceptual difficulty (Williams et al., 2019). More recent studies show that pedagogically integrated robot-based AI programs improve children’s knowledge, skills, and attitudes (Su and Yang, 2023), while an 8-week curriculum indicates that direct instruction with scaffolding enhances knowledge and skills, whereas cooperative play better supports positive learning attitudes (Su et al., 2024).

#### Subtheme 4.2: AI as a support for teachers

With the increasing presence of AI-powered apps, platforms, and devices in early childhood education, teachers report both potential benefits and concerns, including ethical risks and fears of replacement (Lee, 2025; Selwyn, 2019). This reflects a shifting professional identity from content expert to facilitator and coach in AI-supported learning environments.

AI can enhance education by streamlining assessment, improving teaching quality, and enabling personalised instruction through adaptive systems that support engagement, retention, and learning outcomes (Chassignol et al., 2018; Chen et al., 2020; Chiu et al., 2023). While AI-driven approaches may scale access to quality education, optimise learning, and strengthen teacher effectiveness and parental involvement (Hwang et al., 2020; Srinivasan, 2022), concerns remain regarding inaccuracies, bias, and equity (Lo, 2023). Parents also play a key role in supporting children’s learning in rapidly changing technological contexts (Pei, 2025c, 2026), while broader implementation raises issues of accessibility, accountability, and social justice (Luo et al., 2024a).

Despite these potentials, integration of AI with early childhood pedagogy remains limited (Su and Zhong, 2022). Culturally responsive design is therefore essential, ensuring that AI-related learning is grounded in children’s prior knowledge and interests (Yang, 2022). Recent design-based research shows that structured AI activities can enhance children’s understanding of robots while supporting creativity and positive STEM attitudes (Su and Yang, 2023, 2024), with age influencing both creativity and engagement patterns (Roll and Wylie, 2016). Cooperative robot play further supports AI learning when embedded in play-based, interactive pedagogies (Su et al., 2024; NAEYC, 2022; Yang et al., 2024). Overall, AI should augment rather than replace core human dimensions of teaching, including empathy and creativity (Selwyn, 2019).

## Discussion

### RQ1: What core competencies are essential for young children to thrive in an AI-influenced world?

A set of holistic competencies in early childhood education in the AI age has evolved to encompass the integrated development of multiple interrelated domains beyond AI literacy. These holistic competencies provide actionable direction about how to apply child-centered integration of AI in early childhood education (Nikolopoulou, 2025).

Based on the themes and subthemes drawn from the thematic analysis in the findings, Figure 4 visualizes these competencies in five key domains, which are integrated. These include foundational cognitive skills, including literacy and numeracy, social–emotional strengths such as resilience, adaptability, motivation, self-awareness, creativity, critical thinking and theory of mind (Holmes et al., 2019; Trilling and Fadel, 2009; Wellman, 2014; Yang et al., 2024); physical and mental well-being within increasingly digital childhood environments (Dresp-Langley, 2020; OECD, 2025); and AI literacy, equip children to understand AI systems, recognize bias, practice digital ethics, and engage with technology safely and globally (Digital Promise, 2024; Huang et al., 2026; Ng et al., 2021; OECD and European Commission, 2026; Su et al., 2023). These competencies for holistic development also incorporate executive function skills, such as working memory, inhibition, and cognitive flexibility, which serve as foundational infrastructure for social–emotional skills, literacy, and numeracy that support learning across domains and health with lifelong impacts (Diamond, 2013, 2016; Harvard University, 2012; Ibbotson, 2023; Moffitt et al., 2010). All these domains are activated and sustained through positive learning dispositions and approaches to learning, including curiosity, persistence, and collaboration (Carr and Claxton, 2002; Pei, 2025c), which support engagement with literacy and numeracy and shape how children apply and transfer competencies across varied contexts.

**Figure 4:**
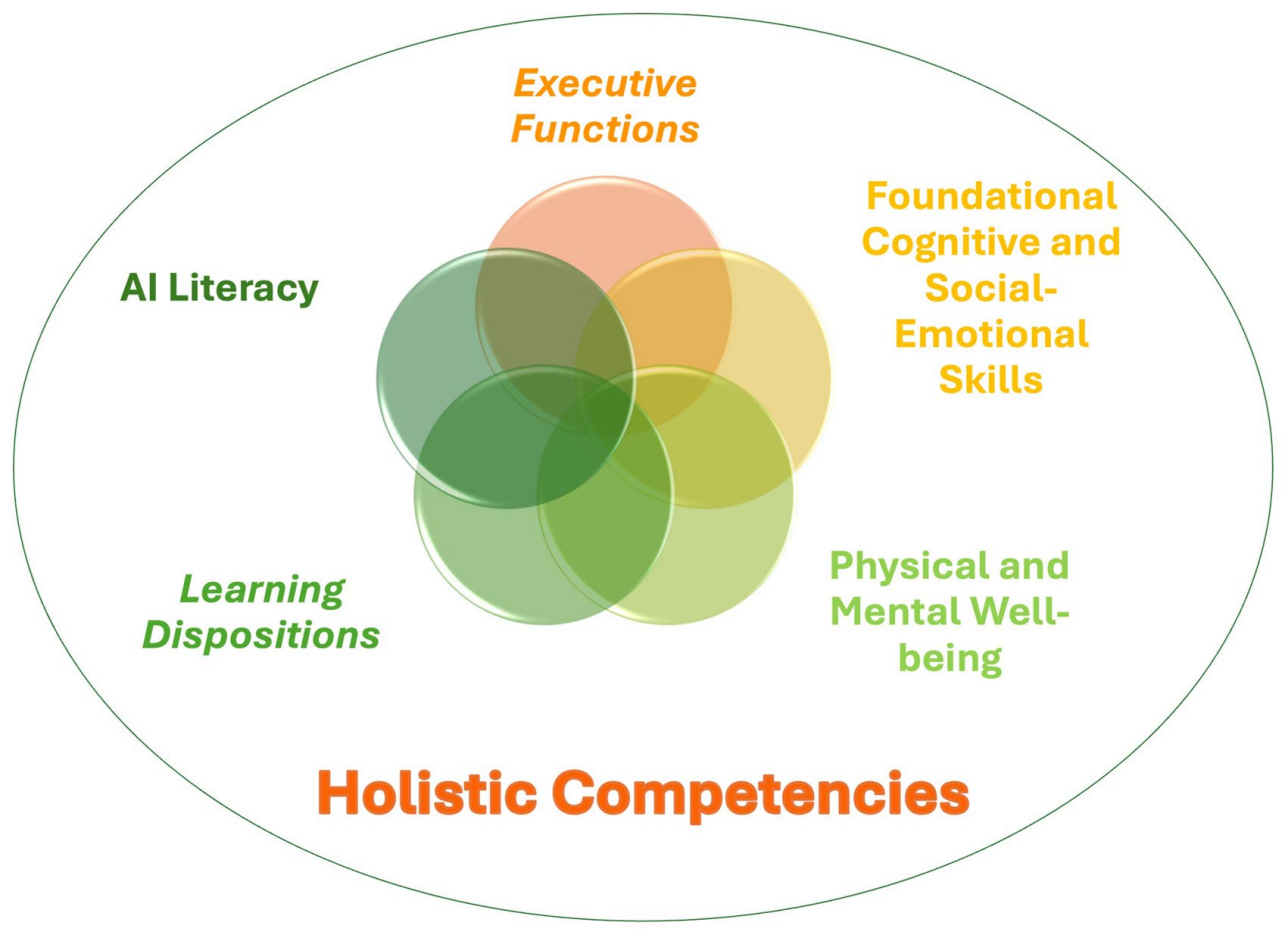
Holistic competencies of young children in the AI age.

These five domains are mutually influential and developed with technical changes at the Chronosystem and influence the societal, cultural, and policy frameworks at the macrosystem (Pei, 2025a). For example, cognitive and social–emotional skills may enhance children’s motivation and confidence to engage with AI tools, while executive function strengthens self-directed learning and supports healthy digital habits. Physical and mental well-being enables sustained attention and cognitive flexibility, which in turn facilitates deeper engagement with learning dispositions and AI literacy. Learning dispositions foster curiosity, initiative, and collaboration, promoting ethical and critical use of technology, while AI literacy informs children’s understanding of digital systems, enhancing decision-making, creativity, and social interactions. Related guidelines are being developed, and more attention should be given beyond AI literacy itself, which, as a new concept that emerged with AI impact, cannot be standalone.

### RQ2: How is AI influencing early childhood educators’ practice, and what supports are needed?

Although literature shows AI tools can support teaching and learning (Chiu et al., 2023; Su and Yang, 2024), early childhood educators face challenges in adopting and integrating them (Chiu et al., 2023; Kölemen and Yıldırım, 2025), this review highlights the need for institutional support at the ecosystem level to boost digital confidence to address the urgent need for transformative knowledge in early childhood education and offer actionable directions for policy and practice (Chen and Li, 2025).

Professional development for educators at the exosystem level is shaped by holistic competencies in the chronosystem and macrosystem (Pei, 2025a), as discussed in RQ1, and influenced by policy guidelines in the macrosystem. AI integration is reshaping educator roles, shifting them from content providers to facilitators, co-designers, and ethical overseers of technology-mediated learning. Educators are required to critically evaluate AI outputs, contextualize them developmentally, and maintain relational pedagogy alongside digital tools (Collie and Martin, 2025; Miao and Cukurova, 2024), incorporating it into holistic curriculum design and thereby generating a ripple effect across the mesosystem. Educators’ professional development should prioritize practical training through hands-on workshops and mentorship programs that support the effective integration of AI tools into teaching (Yurt and Kaşarcı, 2025). Participatory design-based research can effectively support professional learning by actively involving educators in co-design processes and incorporating emerging policy guidelines at the mesosystem level. In addition, early childhood educators always report being overwhelmed by the need to learn new capabilities, thus unplugged activities (Matthews et al., 2025) with systemic support (Pei, 2025b), emerge as a more effective way to empower young children with holistic competencies in the AI age.

At the mesosystem level, collaboration between parents and schools (Pei, 2025a) would benefit from being guided by holistic competencies, ensuring that children receive consistent guidance and support in ethical AI use and holistic development (Srinivasan, 2022).

Direct interactions between children and educators at the microsystem when adopting AI tools should be through play and hands-on experiences (Lee, 2025), ideally, be tangible (Matthews et al., 2025). Play-based learning (NAEYC, 2022; Pei, 2026) can support early AI literacy (Huang et al., 2026; OECD and European Commission, 2026), align with cognitive and social–emotional skills improvement (Holmes et al., 2019), especially meaningful for developing the theory of mind (Wellman, 2014), while appropriate direct instructions with scaffolding (Vygotsky, 1978) also have value for improving some areas, such as conceptual thinking, sequencing, and self-regulation (Su et al., 2024). Given the need for holistic competencies and the nature of young children, curriculum design may integrate early AI literacy across other competency domains, including foundational cognitive and social–emotional skills, physical and mental well-being, executive functions, learning dispositions (Diamond, 2016; Holmes et al., 2019; OECD and European Commission, 2026; Pei 2025c), and connect learning to children’s everyday experiences (NAEYC, 2022; Piaget and Inhelder, 1969; Vygotsky, 1978). This pedagogy will support relational and exploratory learning, preserving essential human elements such as empathy and creativity (Fleer, 2021; Matthews et al., 2025).

In summary, this study advances an ecological synthesis of AI integration in early childhood education by demonstrating how competencies, pedagogical practices, and systemic conditions are co-constructed across interconnected ecological levels Building on the responses to RQ1 and RQ2, Figure 5 visualizes the ecological system for young children’s education in the AI age by synthesising themes generated in this review of 86 resources about AI in early childhood education, as well as other previous studies related to improving early childhood education quality, drawing on Bronfenbrenner’s (1979, 2005) ecological systems theory. Rather than positioning AI literacy as an isolated capability, the findings reconceptualise it as embedded within a broader constellation of cognitive, social–emotional, physical, and dispositional competencies that collectively support children’s adaptive development in AI-mediated environments. This ecological framing contributes to theorising early childhood education in the AI era by linking individual developmental processes with pedagogical practice and policy structures, thereby offering a multi-level account of how education systems respond to technological transformation. Future research should move beyond conceptual synthesis toward empirical and design-based studies that examine how these ecological dynamics operate in practice across diverse early childhood contexts.

**Figure 5:**
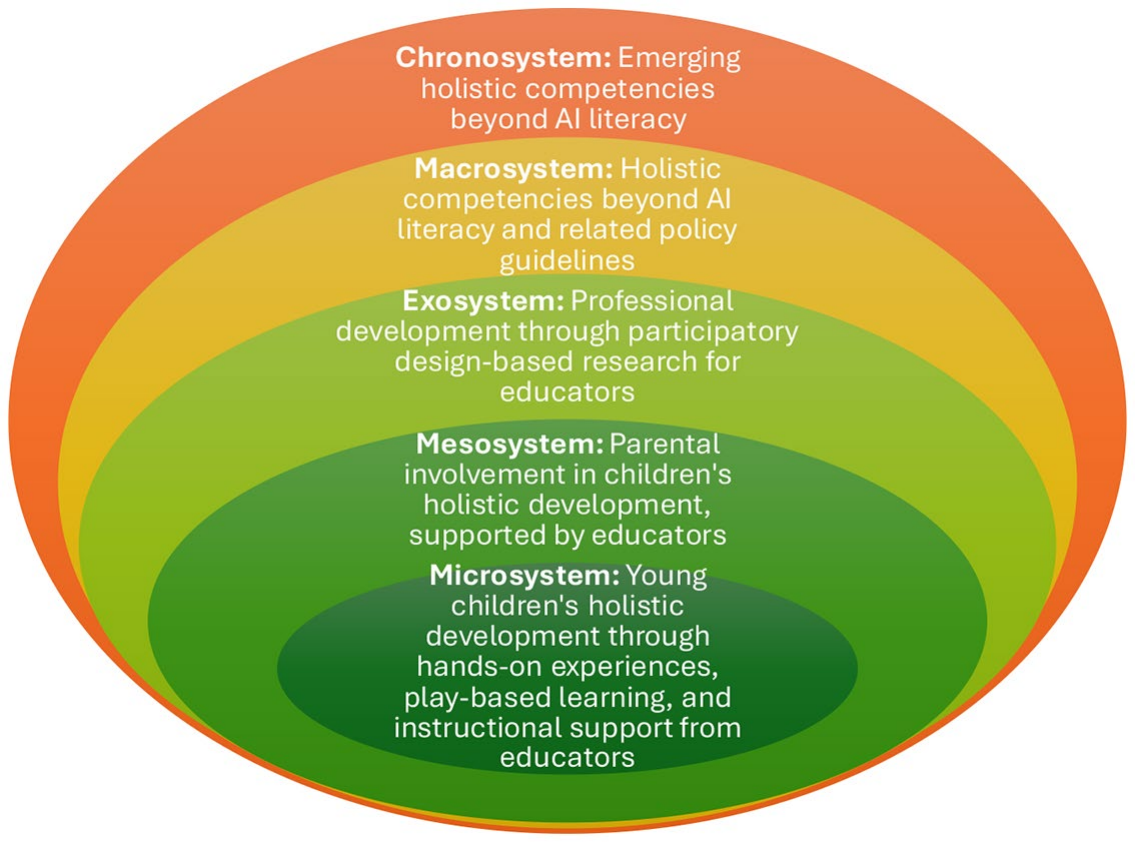
Ecological support for young children’s development in the AI age (Bronfenbrenner, 1979, 2005).

## Conclusion and limitations

This systematic conceptual mapping review highlights the transformative potential of AI in early childhood education. Five interrelated domains of competencies, foundational cognitive and social–emotional skills, physical and mental well-being in the digital age, executive function, learning dispositions and approaches to learning, and AI literacy and technological awareness, were identified, reflecting influences across ecological systems from the chronosystem to the microsystem. Successful integration of AI depends on addressing systemic barriers, including gaps in digital literacy, ethical risks, and pedagogical challenges, as well as the unique pressures faced by female educators, while supporting their evolving roles as facilitators of digital learning and mediators between technology and child development. Realising AI’s potential requires systemic support, including governance structures, targeted teacher professional development, and robust ethical safeguards to ensure developmentally appropriate, equitable, and pedagogically aligned implementation. These findings inform curriculum design by highlighting the need to embed core competencies for holistic development in early childhood programs, ensuring children are prepared to thrive in AI-enhanced learning environments. Limitations include the predominance of studies from high- and middle-income contexts, the rapid evolution of AI technologies, and the interpretive nature of thematic analysis. Future research should therefore move beyond conceptual synthesis towards empirical and design-based studies that examine how ecological dynamics of AI integration are enacted in practice across diverse sociocultural contexts, strengthening the evidence base for theory development and pedagogical innovation in the AI era. In addition, the coding in this review was conducted by a single researcher, and inter-coder reliability was not assessed. To support transparency and consistency, the coding framework was iteratively refined using verbatim excerpts and coding memos throughout the review process. Future research may further enhance the robustness of the coding process by incorporating multiple coders or an intra-coder consistency check.
