# Supplementary material for: Shaping early childhood in the age of AI: holistic competencies, pedagogical practices, and ecological support
Source: Front Psychol. 2026 Oct 21;17:1995020. doi: 10.3389/fpsyg.2026.1995020 (PMC13732525; doi:10.3389/fpsyg.2026.1995020)
Supplement: Supplementary file 1 [file Supplementary_file_1.pdf]

Appendix 1: Data Collection

Reference Number	Full Title of the Paper/Document	Author / Year	Initial Classification
**1**	Umbrella review of artificial intelligence in K-12 education	Huang et al. (2026)	1.1
**2**	AI in ECE: A conceptual review of challenges and opportunities	Su et al. (2023)	1.2
**3**	Artificial Intelligence (AI) in early childhood education: Curriculum design and future directions	Su & Zhong (2022)	1.3
**4**	Artificial intelligence in early childhood education: A scoping review	Su & Yang (2022)	1.4
**5**	When AI Meets Early Childhood Education: Large Language Models as Assessment Teammates in Chinese Preschools	Li et al. (2026)	1.5
**6**	Teachers' AI digital competencies and twenty-first century skills in the post-pandemic world	Ng et al. (2023)	1.6
**7**	Enhancing teachers' technological self-efficacy and well-being: A qualitative study of an "AI for Beginners" professional development program	Gribiea (2026)	1.7
**8**	Conceptualizing AI literacies for children and youth: A systematic review on the design of AI literacy educational programs	Atias & Mawasi (2025)	1.8
**9**	Conceptualizing AI literacy: An exploratory review	Ng et al. (2021)	1.9
**10**	A systematic review of AI literacy conceptualization, constructs, and implementation and assessment efforts (2019–2023)	Almatrafi et al. (2024)	1.10
**11**	Revolutionizing education with AI: Exploring the transformative potential of ChatGPT	Adiguzel et al. (2023)	1.11
**12**	Innovating responsibly: Ethical considerations for AI in early childhood education	Berson et al. (2025)	1.13
**13**	Tracking the Development of Learning Dispositions	Carr & Claxton (2002)	1.14
**14**	Artificial Intelligence trends in education: A narrative overview	Chassignol et al. (2018)	1.15
**15**	Artificial intelligence in education: A review	Chen et al. (2020)	1.16
**16**	Systematic literature review on opportunities, challenges, and future research recommendations of artificial intelligence in education	Chiu et al. (2023)	1.17
**17**	Teachers' early uptake of genAI in teaching and learning: Important questions and answers	Collie & Martin (2025)	1.18
**18**	Executive functions	Diamond (2013)	1.20
**19**	Children's Health in the Digital Age	Dresp-Langley (2020)	1.21
**20**	Exploring the impact of AI on teacher leadership: Regressing or expanding?	Ghamrawi et al. (2024)	1.22
**21**	Executive function and self-regulation: Bi-directional longitudinal associations and prediction of early academic skills	Howard et al. (2021)	1.23
**22**	Vision, challenges, roles and research issues of Artificial Intelligence in Education	Hwang et al. (2020)	1.24
**23**	AI as a substitute for lower-order cognition: Generative AI automates encoding, decoding, and information retrieval	Jiang et al. (2025)	1.25

**24**	The effects of pre-service early childhood teachers' digital literacy and self-efficacy on their perception of AI education for young children	Lim (2023)	1.27
**25**	Generative AI and the future of education: Ragnarök or reformation? A paradoxical perspective from management educators	Lim et al. (2023)	1.28
**26**	What is the impact of ChatGPT on education? A rapid review of the literature	Lo (2023)	1.29
**27**	Aladdin's genie or Pandora's box for early childhood education? Experts chat on the roles, challenges, and developments of ChatGPT.	Luo et al. (2024a)	1.30
**28**	Safety, identity, attitude, cognition, and capability: The 'SIACC' framework of early childhood AI literacy	Luo et al. (2024b)	1.31
**29**	Improving education quality in Chinese universal private preschools	Pei (2025c)	1.32
**30**	Computational thinking tools for early years education- a design study	Matthews et al. (2025)	1.33
**31**	Ethical principles for artificial intelligence in education	Nguyen et al. (2023)	1.35
**32**	Chatbots applications in education: A systematic review.	Okonkwo & Ade-Ibijola (2021)	1.36
**33**	Digital rights, digital citizenship and digital literacy: What's the difference?	Pangrazio & Sefton-Green (2021)	1.37
**34**	Evolution and revolution in artificial intelligence in education	Roll & Wylie (2016)	1.38
**35**	AI & learning: A preferred future	Srinivasan (2022)	1.40
**36**	Artificial Intelligence (AI) literacy in early childhood education: an intervention study in Hong Kong	Su & Yang (2024)	1.41
**37**	AI literacy curriculum and its relation to children's perceptions of robots and attitudes towards engineering and science: An intervention study in early childhood education	Su & Yang (2023)	1.42
**38**	Early artificial intelligence education: Effects of cooperative play and direct instruction on kindergarteners' computational thinking, sequencing, self-regulation and theory of mind skills	Su et al. (2024)	1.43
**39**	Testing longitudinal associations between executive function and academic achievement.	Willoughby et al. (2019)	1.46
**40**	Examining children's perceptions of AI-enabled interactive media characters	Xu et al. (2024)	1.47
**41**	Artificial Intelligence education for young children: Why, what, and how in curriculum design and implementation	Yang (2022)	1.48
**42**	Artificial intelligence education for young children: A case study of technology-enhanced embodied learning	Yang et al.(2024)	1.49

**43**	Suitability of Chinese GenAI platforms for early childhood education: A multifaceted evaluation. AI, Brain & Child, 1(7).	Yu et al. (2025)	1.50
**44**	Persuasion in Human–Artificial Intelligence Systems: How Confidence and Precision Influence Acceptance of Recommendations in a Consumer Context	Chacon & Langer (2026)	1.51
**45**	From stochastic parrots to digital intelligence: The evolution of language models and their cognitive capabilities	Lizarraga et al. (2025)	1.54
**46**	On the dangers of stochastic parrots: Can language models be too big?	Bender et al. (2021)	1.55
**47**	The social neuroscience of empathy.	Singer & Lamm (2009)	1.56
**48**	How common are common mental disorders? Evidence that lifetime prevalence rates are doubled by prospective versus retrospective ascertainment	Moffitt et al. (2010)	1.57
**49**	Embedding local culture in playful early learning: Insights from China via CHAT.	Pei (2026)	1.58
**50**	The development of executive function: Mechanisms of change and functional pressures.	Ibbotson (2023)	1.59
**51**	A new era in early childhood education (ECE): Teachers' opinions on the application of artificial intelligence	Kölemen and Yıldırım (2025)	1.60
**52**	PopBots: Designing an artificial intelligence curriculum for early childhood education.	Williams et al. (2019)	1.61
**53**	Using artificial intelligence (AI)-interfaced robotic toys in early childhood settings: A case for children's inquiry literacy.	Kewalramani et al.(2021)	1.62
**54**	Play with AI (PL-AI): A play-centered, design-based curriculum for AI literacy in pre-K and kindergarten	Lee (2026)	1.63
**55**	Quality early learning: Nurturing children's potential.	Bendini & Devercelli (2022)	2.2
**56**	A pedagogy of play: Supporting playful learning in classrooms and schools.	Ben Mardell et al. (2023)	2.3
**57**	Why improving and assessing executive functions early in life is critical	Diamond (2016)	2.4
**58**	Play in the Early Years	Fleer (2021)	2.5
**59**	The Early Learning and Development Guidelines in China: Retrospective and Prospective Views.	Gao & Huo (2017)	2.6
**60**	Advancing data science education in K–12: Foundations, research, and innovations.	Lee (2025)	2.7
**61**	Open AI in education, the responsible and ethical use of ChatGPT towards lifelong learning	Mhlanga (2023)	2.8
**62**	Developmentally appropriate practice in early childhood programs serving children from birth through age 8	NAEYC (2022)	2.9
**63**	Should robots replace teachers? AI and the future of education	Selwyn (2019)	2.10
**64**	21st Century Skills: Learning for Life	Trilling & Fadel (2009)	2.11
**65**	Making minds: How theory of mind develops.	Wellman (2014)	2.12
**66**	Executive function in preschool-age children: Integrating measurement, neurodevelopment, and translational research.	Griffin et al. (2016)	2.13
**67**	Artificial intelligence in education: Promises and implications for teaching and learning.	Holmes et al. (2019)	2.14

**68**	Artificial intelligence methods in early childhood education	Prentzas, J. (2013)	2.15
**69**	Australian Framework for Generative Artificial Intelligence in Schools.	Department of Education (2023)	3.2
**70**	Building the brain's "air traffic control" system: How early experiences shape the development of executive function.	Harvard University (2011)	3.4
**71**	Executive function: Skills for life and learning	Harvard University (2012)	3.5
**72**	The effects of AI on the working lives of women.	Collett et al. (2022)	3.6
**73**	The Head Start path to positive child outcomes	Department of Health and Human Services (2003)	3.9
**74**	Belonging, being, and becoming: The Early Years Learning Framework for Australia	Department of Education, Employment and Workplace Relations. (2009).	3.10
**75**	AI literacy: A framework to understand, evaluate, and use emerging technology	Digital Promise (2024, June 18)	3.11
**76**	Intelligence unleashed: An argument for AI in education	Luckin et al. (2016)	3.16
**77**	MOE outlines 2025 national strategy for digital education	MOE (2025, April 3)	3.20
**78**	Philanthropy for social and emotional learning	OECD (2023)	3.25
**79**	Empowering learners for the age of AI: An AI literacy framework for primary and secondary education	OECD & European Commission (2026)	3.27
**80**	How's Life for Children in the Digital Age?	OECD (2025b)	3.28
**81**	Artificial intelligence and the future of teaching and learning	Office of Educational Technology (2023)	3.31
**82**	The future of educational technology	Stanford University (2024)	3.32
**83**	Gender equality in and through the teaching profession	UNESCO (2023, November 8)	3.38
**84**	Guidance for generative AI in education and research	Miao & Holmes (2023)	3.39
**85**	Five big ideas in artificial intelligence	AI4K12 Initiative (2024)	3.40
**86**	AI competency framework for teachers.	Miao & Cukurova (2024)	3.43
